# A disposable, ultra-fine endoscope for non-invasive, close examination of the intraluminal surface of the peritoneal dialysis catheter and peritoneal cavity

**DOI:** 10.1038/s41598-020-74129-2

**Published:** 2020-10-16

**Authors:** Masaaki Nakayama, Chieko Hamada, Keitaro Yokoyama, Yudo Tanno, Nanae Matsuo, Junichiro Nakata, Yoshio Ishibashi, Atsushi Okuzawa, Kazuhiro Sakamoto, Tamaki Nara, Takatoshi Kakuta, Masaomi Nangaku, Takashi Yokoo, Yusuke Suzuki, Toshio Miyata

**Affiliations:** 1 Medical Technology Innovation Center, Juntendo University, Bunkyo-ku, Hongo 2-1-1, Tokyo, 113-8421 Japan; 2 United Centers for Advanced Research and Translational Medicine (ART), Tohoku University Graduate School of Medicine, Sendai, Japan; 3 Kidney Center, St Luke’s International Hospital, Tokyo, Japan; 4 Division of Nephrology, Department of Internal Medicine, Juntendo University Faculty of Medicine, Tokyo, Japan; 5 Division of Nephrology and Hypertension, Department of Internal Medicine, The Jikei University School of Medicine, Tokyo, Japan; 6 Department of Surgery, The Jikei University School of Medicine, Tokyo, Japan; 7 Department of Coloproctological Surgery, Juntendo University Faculty of Medicine, Tokyo, Japan; 8 Division of Nephrology and Metabolism, Tokai University Hachioji Hospital, Tokyo, Japan; 9 Division of Nephrology and Endocrinology, The University of Tokyo, Tokyo, Japan

**Keywords:** Nephrology, Engineering

## Abstract

The ability to visualize intraluminal surface of peritoneal dialysis (PD) catheter and peritoneal cavity could allow elucidation of the cases of outflow problems, and provide information on changes to the peritoneal membrane leading to encapsulating peritoneal sclerosis. A non-invasive examination that allows those monitoring in need is desirable. We have developed a disposable ultra-fine endoscope that can be inserted into the lumen of the existing PD catheter, allowing observation of the luminal side of the catheter and peritoneal cavity from the tip of the PD catheter, with minimum invasion in practice. In a pre-clinical study in pigs and a clinical study in 10 PD patients, the device provided detailed images, enabling safe, easy observation of the intraluminal side of the entire catheter, and of the morphology and status of the peritoneal surface in the abdominal cavity under dwelling PD solution. Since this device can be used repeatedly during PD therapy, clinical application of this device could contribute to improved management of clinical issues in current PD therapy, positioning PD as a safer, more reliable treatment modality for end-stage renal disease.

## Introduction

Peritoneal dialysis (PD) is a home-based renal replacement therapy that can support the social rehabilitation of patients with end-stage renal failure. PD is cost-effective and could be the first-line dialysis modality. However, technical failure due to catheter-related problems and the development of encapsulating peritoneal sclerosis (EPS) have been critical issues in clinical practice.

Mechanical complications of the PD catheter, such as intraluminal clogging by omentum, fibrin, or blood clot, cause out and inflow problems, and are major causes of poor catheter survival^1,2^. The ability to obtain intraluminal images from the PD catheter could allow prompt elucidation of the causes, facilitating adequate management^3^.

PD vintage is connected with morphological changes of the membrane during PD therapy, and an increased incidence of EPS^4^. As a result, limits to the time on PD have been discussed^5^. However, several recent studies have indicated that the use of neutral solutions with low glucose degradation products can preserve the histology of peritoneal tissue^6–8^. Furthermore, improvements in the biocompatibility of the dialysis solution have reportedly decreased the incidence of EPS^9^. Such observations raise questions regarding the scientific/medical rationales underpinning time limits on PD in general. The ability to visualize the peritoneal membrane could provide key patient-specific information, and guide decisions on the safety of continuing PD. Non-invasive examination of the intraluminal catheter and peritoneal membrane during the course of PD treatment could thus have enormous clinical benefit.

To address these issues, we have developed a non-invasive, ultra-fine endoscope specifically for use in patients on PD. This novel device can be used to repeatedly examine the intraluminal side of the catheter and surface of the peritoneum without needing surgery or anesthesia in the patient. Here, we report the details of this device and results from our pre-clinical and clinical studies in 10 patients on stable PD.

## Results

No events such as bleeding or organ injuries occurred in either pre-clinical or clinical studies, and no symptoms such as pain needing anesthesia or peritonitis were observed in the clinical study. There were no catheter and exit-site complications during the insertion or after the endoscope was removed. In the clinical study, no prophylaxis antibiotics for the procedure were used in all cases. In the pre-clinical study of two pigs, we examined the potential risk of peritoneal injury through maneuvering the endoscope as follows; the endoscope was projected from the tip of catheter and strongly scratched the surface of parietal peritoneum under the condition with or without PD solution. As a result, no injuries were observed in all cases. As shown in the fluoroscopy video of the pre-clinical study (S1), the free part of the endoscope which is projected from the tip of PD catheter may move momentarily. Although we did not find any adverse events in this case (pig), we decided that the tip of the endoscope and guiding-catheter should not be projected from the tip of the PD catheter in order to prevent un-predictable movement of the tip of the endoscopy, which could be the risk of the peritoneal damage. Based on this regulation, no problems were noticed in regard to maneuverability of the device in any studies.

**Figure 1:**
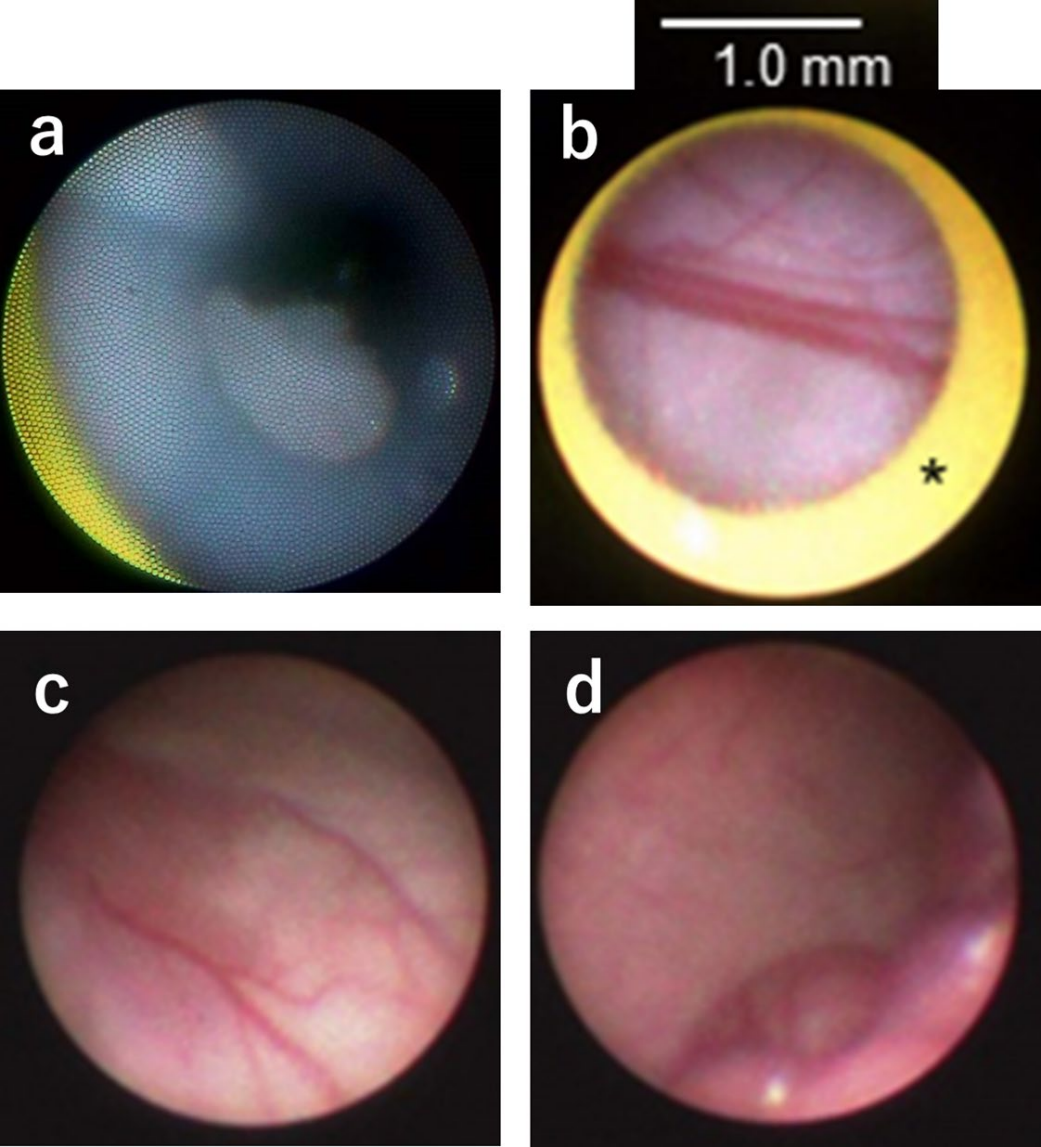
Endoscopic findings for the intraluminal side of the PD catheter and peritoneal surface using the PD endoscope in pigs (Supplementary S1: online). (**a**) Close-up image of omental extrusion through a side hole in the PD catheter. (**b**) Close-up image of peritoneal blood vessels on the visceral surface (capillaries, small and medium-sized veins). (**c**) Distant image of peritoneal blood vessels in the parietal wall. (**d**) Distant image of the parietal (upper) and visceral (lower) surface. In close-up images, approximate length of the image can be estimated by the lumen diameter of the guiding catheter tip. *Intra-luminal portion of the guiding catheter (yellow portion).

Endoscopic images: The color tone of the images obtained using this novel endoscope under dwelling PD solution was equivalent to that obtained with the currently used rigid laparoscope under air in pigs. The condition of the PD catheter lumen was clearly observable, and omentum that had entered into the PD catheter through the side holes (Fig. 1a). On close-up images (approximately 10 mm from object to endoscope tip), differences in the color and thickness of vessels (i.e., capillaries and venules) were easily distinguished (Fig. 1b). Distant images (within approximately 800 mm from object to endoscope tip) enabled observation for the surface of the parietal and visceral walls (Fig. 1c,d).

**Figure 2:**
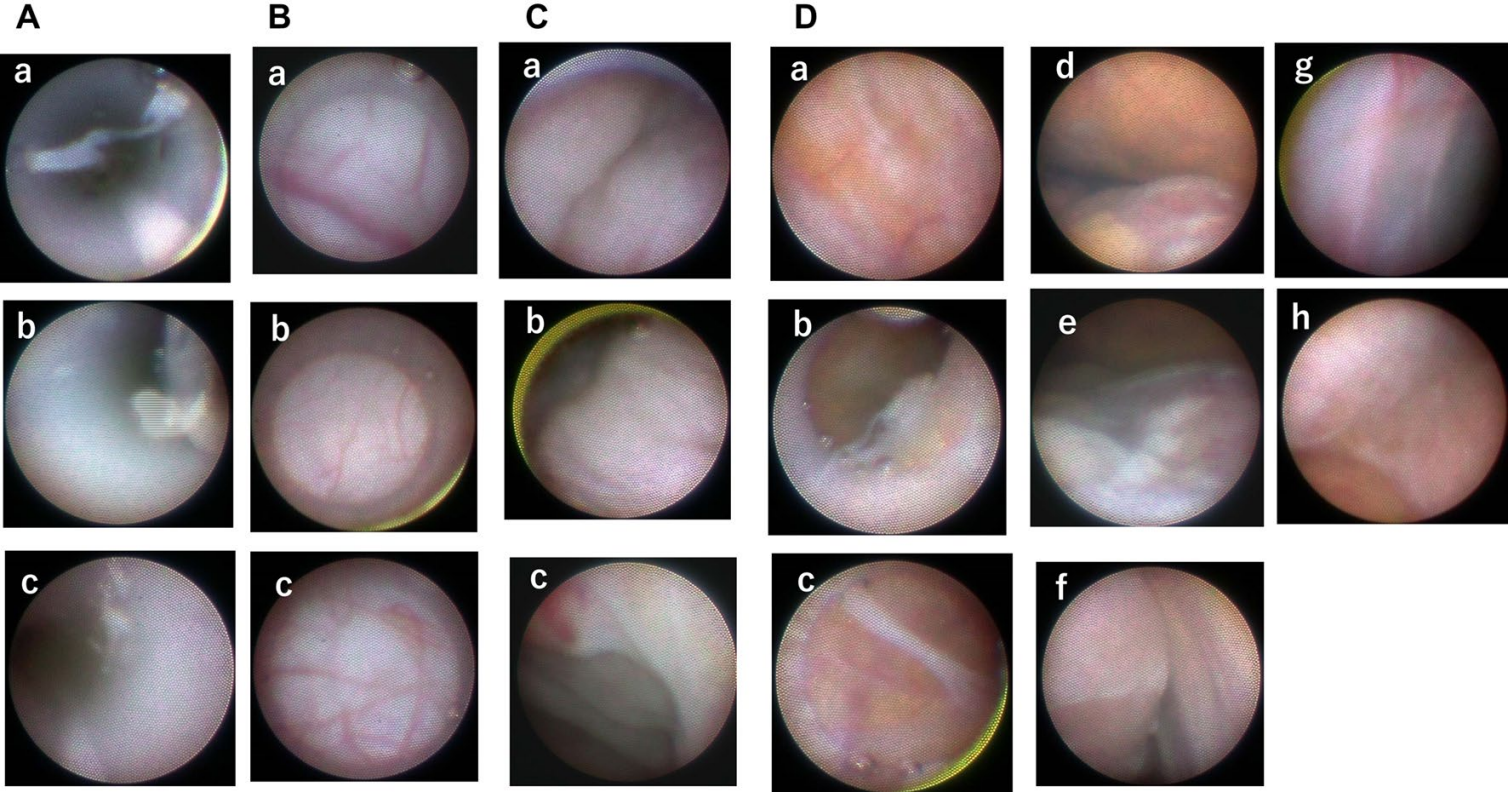
Endoscopic findings for the intraluminal side of the PD catheter and peritoneal surface using the PD endoscope in patients on PD. (**A**) Intraluminal side of the PD catheter: Fibrin fragment in the catheter lumen in three patients, fibrin aggregation sticking to the lumen (a) (Supplementary S2 online), and fibrin floating in the lumen (b, c). (**B**) Microvessels: Close-up image of veins and post-capillary venule of the parietal wall (a), capillaries of the visceral membrane surface (b), and spider-like winding microvessels on the visceral membrane (c). (**C**) Parietal and visceral walls: Distant image of the visceral surface with no marked findings (a, b), and surface of the visceral peritoneum with spotted small ecchymosis (c). (**D**) Fibrin deposits and membrane formation in three cases: Case No. 3, 64 years old, 4 months on PD, underlying kidney disease; diabetic nephropathy. Patchy white color changes noted on the parietal wall (a), aggregated fibrin clot on the visceral surface (b), and presence of aggregated fibrin bridging both sides of the parietal wall and intestine surface (c). Case No. 8, 70 years old, after 42 months on PD, underlying kidney disease; IgA nephropathy (Supplementary S3 online). Surface of the parietal (upper) and visceral (lower) peritoneum. Presence of translucent membrane with patchy white fibrin mass (d). Surface of the intestine is visible through a gap in the fibrin mass on the translucent membrane (e). Surfaces of the parietal (Rt) and visceral (Lt) peritoneum. Fibrin sheet is present on part of visceral surface (Lt upper side*) (f). Case No. 9, 66 years old, after 65 months on PD, underlying kidney disease; IgA nephropathy. Translucent membrane is present covering the original parietal membrane (g), visceral (Lt upper) and parietal surface with membrane extending on both sides (h). Endoscopic images (Figs. 1, 2) reflected by camera (3CMOS HD Camera FC-304: FiberTec Co., Ltd. Japan) were edited by Adobe Premiere Elements 2020 (https://www.adobe.com/jp/products/premiere-elements).

In the clinical study in 10 PD patients (Table 1), the condition of the PD catheter lumen was clearly observable, and microvessels were easily distinguished in all cases. The presence of fibrin in the lumen of PD catheter was identified in three cases, and unique changes of the peritoneal membrane could be identified, such as vascular changes in two cases, isolated fibrin deposits on the membrane in six cases, and localized/extended encapsulating membrane which was not adhered to the intestine in three cases (Fig. 2). There were no interventional procedures performed during the endoscopic examinations. As to the fibrin in the lumen in three cases, the characteristics of fibrin clots were all soft and fragile, and easily removed by flushing of PD solution. Mobility of the intestine could be observed well enough to identify the adhesive lesion between encapsulating membrane and intestine.

**Table 1:** Patients demographics and summary of endoscopic findings.

No.	Gender	Age (years)	PD vintage (months)	Modality	Underlying kidney disease	PET	Number of peritonitis history	Endoscopic findings presence/detection of ① mocrovessles, ② morphologic change of local vasculature, ③ localized (L) or extended (E) membrane formation, ④ localized fibrin exudate/deposit in peritoneum, and ⑤ fibrin clot/fragment in the intraluminal side of catheter				
								①	②	③	④	⑤
1	Male	63	40	PD	IgAN	H	None	○			○	
2	Male	66	27	PD	CGN	H	None	○	○		○	○
3	Male	64	4	PD	DKD	HA	None	○			○	○
4	Male	48	19	PD	DKD	LA	None	○		○ (L)	○	○
5	Male	51	9	PD	DKD	LA	None	○			○	
6	Male	61	77	PD + HD	DKD	HA	1	○				
7	Male	56	36	PD	Unknown	LA	None	○				
8	Male	70	42	PD	IgAN	L	1	○		○ (E)	○	
9	Male	66	65	PD + HD	IgAN	HA	1	○		○ (L)		
10	Female	53	39	PD + HD	Unknown	LA	1	○	○			

*PD* peritoneal dialysis, *HD* hemodialysis, *PD + HD* combination therapy of PD and HD, *CGN* chronic glomerulonephritis, *DKD* diabetic kidney disease, *IgAN* IgA nephritis, *PET* peritoneal equilibration test, *H* high, *HA* high average, *LA* low average, *L* low

## Discussion

This non-invasive, ultra-fine endoscope was developed to observe the status of the lumen of the PD catheter and the peritoneal membrane surface in patients on PD. Our pre-clinical and clinical studies encountered no adverse events during maneuvering of the device, and the risk of tissue injury from the endoscope in the presence of dialysis solution seemed low.

The high-quality imaging of the catheter lumen could detect invasion of omentum into the lumen through the side holes in the pre-clinical study, and provided information on fibrin deposits in the clinical study. Thus, close observation of the catheter lumen is expected to allow easier clarification of the specific causes of catheter obstruction and better checking for abnormalities within the catheter lumen. In terms of actual clinical utility, this device may allow observation of the formation of biofilm, which is a risk factor for recurrent peritonitis. In cases showing biofilm formation, catheter removal is currently mandatory. However, the appearance and process of biofilm formation in cases with PD catheter-related peritonitis has remained a "black box" in clinical settings, and the present device may help answer key questions regarding this phenomenon.

Currently, laparoscopy is the only modality that allows direct visual observation of the PD peritoneum. Recent studies in Japan have started to investigate relationships between morphology and function of the peritoneum in patients undergoing PD using a commercially available laparoscope^10–13^. With that device and approach, changes in the peritoneum during PD, such as fibrin deposition, and capsule formation, which are assumed to represent prodromes of EPS, can be evaluated clinically. However, the laparoscopic approach currently used is highly invasive and needs to be performed under local or general anesthesia by highly skilled doctors, so its suitability for repeat examination is limited. In many cases, such observations are only performed at the time of catheter removal when PD is discontinued^10–13^, and the current laparoscopy is thus not ideally situated for assessing PD continuation. In our pre-clinical study, close-up images of the peritoneal surface observed using this device were satisfactory from a clinical perspective when compared with those obtained using existing rigid laparoscopes. Similarly, sufficiently detailed images of the peritoneal membrane in terms of vasculature, fibrin deposition, and encapsulating membrane were provided in the clinical study. Repeat observation of the peritoneum in clinical settings would be clinically useful for PD specialists for determining consensus findings of peritoneal deterioration based on temporal changes in peritoneal features. The advent of an innovative non-invasive medical device that allows assessment of peritoneal tissue and early detection of EPS may have enormous clinical benefits. Furthermore, this device may also be used to visually examine the peritoneum for other purposes, such as assessing new drugs to protect the peritoneum or the biocompatibility of new dialysis solutions. As to the limitations of this device, since the present device was developed only to observe objects, there is no capability in respect to the therapeutic interventions, which are different from rigid-type current laparoscope. In future, development of novel functions, such as, capabilities to collect tissue samples, resect tissue adhesions, and identify details of microvessels, would be expected. Secondly, due to the catheter rigidity, this endoscope may not be applicable for coiled catheters. And lastly, disposing the endoscope after each use may not make this procedure cost effective. These issues need to be addressed.

Use of this device will help to position PD as a safer, more reliable treatment modality for patients with end-stage renal failure. This novel endoscopic device may have major impacts on the clinical practice of PD.

## Materials and methods

### Structure of the device

**Figure 3:**
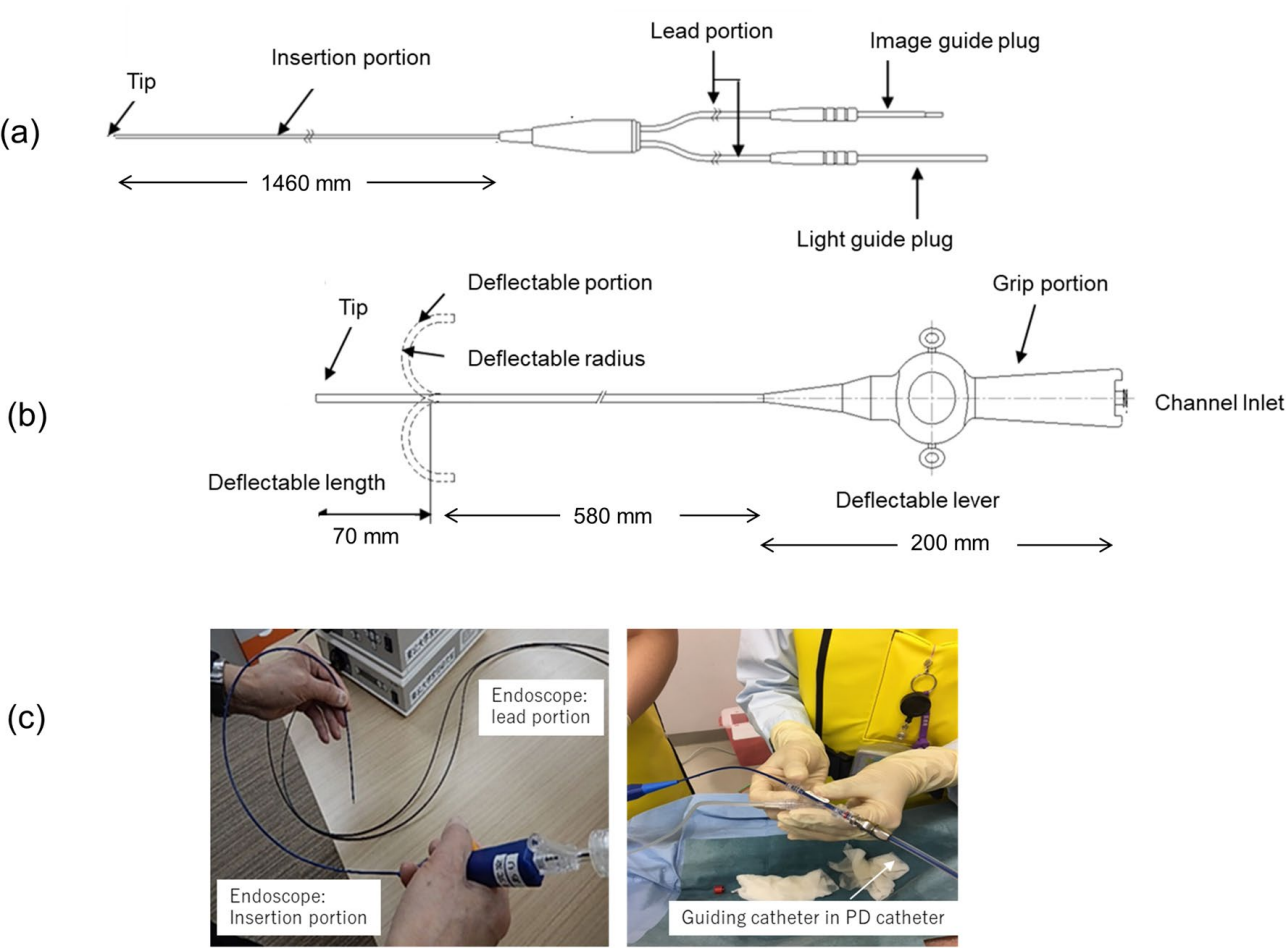
Structure of the disposable ultra-fine endoscope for PD. Details of the ultra-fine endoscope (**a**) and deflectable guiding catheter (**b**), and images of endoscopic manipulation (**c**). The inserted portion of the main endoscope and the deflectable guiding catheter have an external hydrophilic coating to facilitate smooth passage into the lumen of the deflectable guiding catheter and lumen of the PD catheter. Figures were drawn by AUTOCAD (https://www.autodesk.co.jp/products/autocad/overview).

The prototype of this medical device was originally developed in Japan as a highly versatile, fine-diameter endoscope. We modified and optimized the device specifically for use in patients on PD. The endoscope was developed for single use in terms of the sterilization resistance. The device essentially has two main parts: an endoscope, and a guiding catheter. The endoscope itself is a disposable, flexible laparoscope (Fig. 3a) with an inserted portion that has an outer diameter of 1.3 mm. The deflectable guiding catheter (Fig. 3b) has a lumen diameter of 1.8 mm and an outer diameter of 2.3 mm. The endoscope inside the deflectable guiding catheter lumen is passed through the indwelling PD catheter (inner diameter, 2.6 mm) to facilitate non-invasive entry into the peritoneal cavity (Fig. 3c). The inserted portion of the laparoscope can be passed through the lumen of the guiding catheter, and the range of the endoscopic field of view can be expanded by manipulating the deflectable tip of the guiding catheter in the peritoneal cavity using a lever at the operator's end. The instrument was designed under the premise that examination would be conducted in a liquid environment of a lumen filled with PD solution, thereby allowing examination to be performed without interrupting dialysis treatment.

### Pre-clinical and clinical studies

The research protocols for the pre-clinical and clinical evaluations of this instrument were designed at Tohoku University (Sendai, Japan). The pre-clinical study was performed between September 2016 and February 2017 at Juntendo University (Tokyo, Japan), and the clinical study was conducted between April 2018 and July 2018 at Juntendo University and The Jikei University (Tokyo, Japan). The pre-clinical study was approved by the Experimental Animal Committee at Juntendo University Faculty of Medicine (2016/07/19, approval no. 280257). The clinical study was approved by the ethics committee at Juntendo University Faculty of Medicine (2017/12/01, approval no. 2017-025) and at The Jikei University (2018/01/23, approval no. 29-2(52)). Informed consent was obtained from all patients. All the experiment protocol for using animals were carried according to relevant guidelines and regulation of animal care. All the experiment protocol for involving humans was in accordance with relevant national/international/institutional guidelines and regulations.

In the pre-clinical study, maneuverability and safety of the device and the quality of images obtained under dwelling PD solution were examined in two 3-month-old 40-kg female pigs. Under general anesthesia with ketamine, the animals were maintained on artificial respiration with endotracheal intubation, and a PD catheter (J1; HAYASHIDERA MEDINOL Co. Ltd., Kanazawa, Japan) was inserted into the peritoneal cavity via the midpoint of the lower abdominal wall. The first cuff was sutured to the peritoneum, the second cuff was placed subcutaneously, and an outlet was created in the upper abdomen to the left of the umbilicus. After observation using the endoscope, each animal was euthanized by administration of pentobarbital.

In the clinical study, the primary purpose was to examine the feasibility of this device in clinical practice. Ten patients on regular PD (age; 60 ± 7 years old, median PD vintage; 37 months [9–71], male; nine cases, underlying kidney disease; diabetic nephropathy in four, chronic glomerulonephritis in four, and unknown in two cases, all on neutral PD solution w/wo icodextrin solution, no episodes of peritonitis during the preceding 3 months) were tested. Three cases among them had been treated by PD and hemodialysis (HD) combination therapy, which is the common modality in Japan; 5 days on PD and once on HD per week^14^. At the examination, after complete drainage of PD solution, 1.5–2.0 L of fresh PD solution was infused. The endoscope and guide catheter were then inserted through the PD catheter in a supine position without using anesthesia. After examination, all patients were followed-up for 7 days with regard to the occurrence of adverse events.

## Supplementary information

Supplementary Information.
