# Supplementary material for: A disposable, ultra-fine endoscope for non-invasive, close examination of the intraluminal surface of the peritoneal dialysis catheter and peritoneal cavity
Source: Sci Rep. 2020 Oct 16;10:17565. doi: 10.1038/s41598-020-74129-2 (PMC7567793; doi:10.1038/s41598-020-74129-2)
Supplement: Supplementary file 1 — Supplementary Information. [file 41598_2020_74129_MOESM1_ESM.docx]

Supplementary data

S-1 Pre-clinical study in pig

https://youtu.be/PqQfVOtXDGY

S-2 Clinical study (Case No.2)

https://youtu.be/E3RbozBD1z0

S-3 Clinical study (Case No.8)

https://youtu.be/EDfwe-Mtff8
